# The early warning and response systems in Syria: A functionality and alert threshold assessment

**DOI:** 10.1016/j.ijregi.2024.100563

**Published:** 2025-01-07

**Authors:** MHD Bahaa Aldin Alhaffar, Aula Abbara, Naser Almhawish, Maia C. Tarnas, Yasir AlFaruh, Anneli Eriksson

**Affiliations:** 1 Department of Global Public Health, Karolinska Institute, Stockholm, Sweden; 2 Diseases Program, European Center for Disease Prevention and Control (ECDC), Stockholm, Sweden; 3 Syria Public Health Network, England, UK; 4 Department of Infection, Imperial College, London, UK; 5 Department of Population Health and Disease Prevention, University of California Irvine, Irvine, USA; 6 Assistance Coordination Unit, Gaziantep, Turkey

**Keywords:** Early warning system, EWARN, EWARS, Syria, Alert threshold, Syndromic surveillance, conflict

## Abstract

**Objectives:**

this study aims to provide an updated evaluation of the functional characteristics of the two Early Warning Systems (EWS) in Syria, EWARS (Early Warning, Alert, and Response System) and EWARN (Early Warning, Alert, and Response Network), and to test different alert threshold methods using World Health Organization guidelines against the data of selected diseases.

**Methods:**

A retrospective analysis of EWARN and EWARS surveillance data assessed functional characteristics. The World Health Organization alert thresholds for measles, acute bloody diarrhea, acute jaundice syndrome, and severe acute respiratory infections were tested using three methods. Sensitivity, specificity, and Youden index determined threshold suitability for each syndrome.

**Results:**

The annual average number of reported cases was 1,140,717 for EWARS and 10,189,415 for EWARN. This study found that the optimal alert thresholds varied among different diseases. The percentile method showed promising results with good sensitivity and specificity. For measles, the 85^th^ percentile threshold had the best results (Youden index = 0.443), whereas for acute bloody diarrhea, it was 75^th^ percentile (Y = 0.532) and for severe acute respiratory infections, it was 90^th^ percentile (Y = 0.653).

**Conclusions:**

This study supports the use of adaptable disease-specific alert thresholds such as the percentile approach. Further research is required to develop statistical methods that can be applied to various early warning systems in conflict contexts.

## Highlights

• Surveillance in conflict is important, challenging, and requires careful examination.
• Limited research was conducted to improve early warning systems in conflict settings.
• Disease-specific thresholds can enhance outbreak alert sensitivity.
• Further research is essential to improve early warning systems in crisis settings.

## Introduction

The Syrian conflict, which began in 2011, has led to one of the gravest humanitarian crises of the 21st century [1], severely impacting health care infrastructure and public health systems [2].

Amidst violence and chaos, Syria's health care infrastructure has been severely damaged [3], hospitals and medical facilities have been directly targeted [4], health care workers have been killed or forced to flee [5], and essential medical supplies have become scarce [6]. This destruction has made the population highly vulnerable to health risks, including the spread of infectious diseases [7]. The breakdown of public health services has hampered efforts to detect, monitor, and control outbreaks, further compounding an already dire humanitarian crisis [8].

This conflict has contributed to the emergence and reemergence of many infectious diseases, some of which had been previously controlled or eliminated, such as poliomyelitis, which reemerged in 2013, highlighting the severe public health challenges posed by the conflict [7]. Increased measles cases, leishmaniasis and the COVID-19 pandemic have further strained the already weak health care system [[9], [10], [11]]. The neglect and interference with water and sanitation contributed to a large cholera outbreak which originated in Aleppo in 2022 but particularly affected northeast (NES) and northwest (NWS) Syria. These outbreaks underscore the urgent need for effective disease surveillance and response mechanisms in such conflict humanitarian crises [9,[12], [13], [14]].

In this challenging context, Early Warning Systems (EWSs) are crucial. Designed as rapid response tools for epidemic-prone diseases during humanitarian crises, EWSs are tailored to function effectively amid the chaos and limited resources inherent in conflict zones [[15], [16], [17]]. EWS aims to rapidly detect, verify, and respond to health threats, emphasizing the need for prompt action to prevent large-scale outbreaks [18,19]. Unlike routine surveillance systems, which focus on long-term health trends, EWSs are designed to rapidly detect and respond to potential outbreaks in emergency settings. Evaluating their effectiveness is crucial for enhancing their outbreak detection capabilities, particularly in conflict-affected regions. The first implementation of an EWS was in 1999 in Sudan (currently South Sudan) in response to an outbreak of relapsing fever that followed the civil war in the country [20]. EWSs have also been implemented in Afghanistan, Iraq, Libya, Yemen, and Syria to respond to public health challenges following humanitarian crises [21].

In Syria, two EWSs were implemented due to the geopolitical situation: the Early Warning, Alert, and Response System (EWARS) under the Ministry of Health with World Health Organization (WHO) support in 2012 [22], and the Early Warning, Alert, and Response Network (EWARN) managed by the Assistance Coordination Unit (ACU) since 2013, with backing from the Gates Foundation and US Centers for Disease Control and Prevention [23]. These EWSs continued to function until November 2024. However, their role as of 8th December 2024, when opposition forces toppled the Syrian regime, is being discussed and reformed given the merger of Syria’s geography under a new transitional government. It is likely that they will merge to provide coverage under a single EWS, though the details of this are yet to be finalised. This change in Syria makes the historic exploration of the two systems within Syria’s borders even more pertinent as EWARS mostly functioned in former Syrian regime held areas and benefited from better resources and support, easing data collection and preventive actions [11]. In areas outside of the previous regime's control, EWARN faced challenges including restricted health care access, complex aid delivery, security issues, and limited resources, which hinder disease detection and response [8,19]. Studies have used EWARN data to assess conflict impacts on measles, diarrheal diseases, and respiratory illnesses, including COVID-19 [10,[24], [25], [26]].

Despite the complexity of EWSs and their differences from routine surveillance, few studies have evaluated their effectiveness. Mala et al. [21] found that EWSs have high completeness and timeliness in emergencies, but the population covered was low, and Dureab et al. [27] assessed EWSs and outbreak responses in Yemen and found that the quality of data and timeliness are the main challenges to the system. A key aspect of EWSs is their alert thresholds, which influence the system's sensitivity and specificity in detecting outbreaks. Research by Rguig et al. [28] on influenza in Morocco and Wang et al. [29,30] on the China Infectious Disease Automated Alert and Response System (CIDARS) in China showed that adjusting the alert thresholds improved outbreak detection. However, these studies focused on routine surveillance, and no research has specifically evaluated the EWARN and EWARS outbreak alert thresholds based on WHO guidelines.

The alert thresholds set by the ACU for EWARN in Syria differ from the WHO guidelines in certain aspects. Although the WHO guidelines suggest thresholds based on fixed values or statistical methods [16,17], the EWARN alert thresholds in Syria are adjusted based on the epidemiological context and expert judgment in managing the system. Since 2018, over 500 alerts have been registered in the areas covered by EWARN, some of which have been declared as outbreaks, such as measles [10], severe acute respiratory infection (SARI), and acute bloody diarrhea (ABD) [26]. However, many of the registered alerts could be false alerts due to the method used to set the alert threshold. Therefore, assessing the alert threshold is important to improve the ability of the system to identify alerts and reduce false notifications.

Our study aims to provide an updated evaluation of the functional characteristics of EWARS and EWARN in Syria using data between 2012 and 2023, and to test different alert threshold methods using the WHO guidelines against the data of selected diseases and the currently used threshold. The findings of this study contribute to the existing body of knowledge on EWSs in different countries and provide methods of alert threshold adjustment that could improve EWS capabilities in responding to outbreaks in complex settings, such as the case of Syria. It will also inform practices relevant to EWS in Syria given the likely merger of the two systems due to the recent geopolitical changes or the formation of a more formal public health surveillance system.

## Methodology

### Study design

In the first stage, we performed a retrospective descriptive analysis of the functional characteristics (including completeness, timeliness, number of sentinel sites, coverage, and types and numbers of surveyed and reported cases) of EWARS (2012-2023) and EWARN (2015-2023). The reporting of the study adhered to the RECORD guidelines [31]. In the second stage, we applied WHO-defined alert thresholds to cases of measles, ABD, acute jaundice syndrome (AJS), and SARI reported by EWARN across four districts in 2023 [16,17].

### Study setting and population

Syria's conflict rapidly escalated into a multifaceted war involving many actors and proxies [10]. The dynamic nature of Syria's political situation has led to multiple shifts in geopolitical control, which, in turn, has affected the coverage of EWARN and EWARS. Until late 2024, Syria's geopolitical landscape was divided into three main areas of control: NES, NWS, and government-held territories, with other smaller regions controlled by different parties [26]. NES and NWS were non–government-controlled areas. These geopolitics are now shifting with the fall of the Assad regime in December 2024.

**Figure 1:**
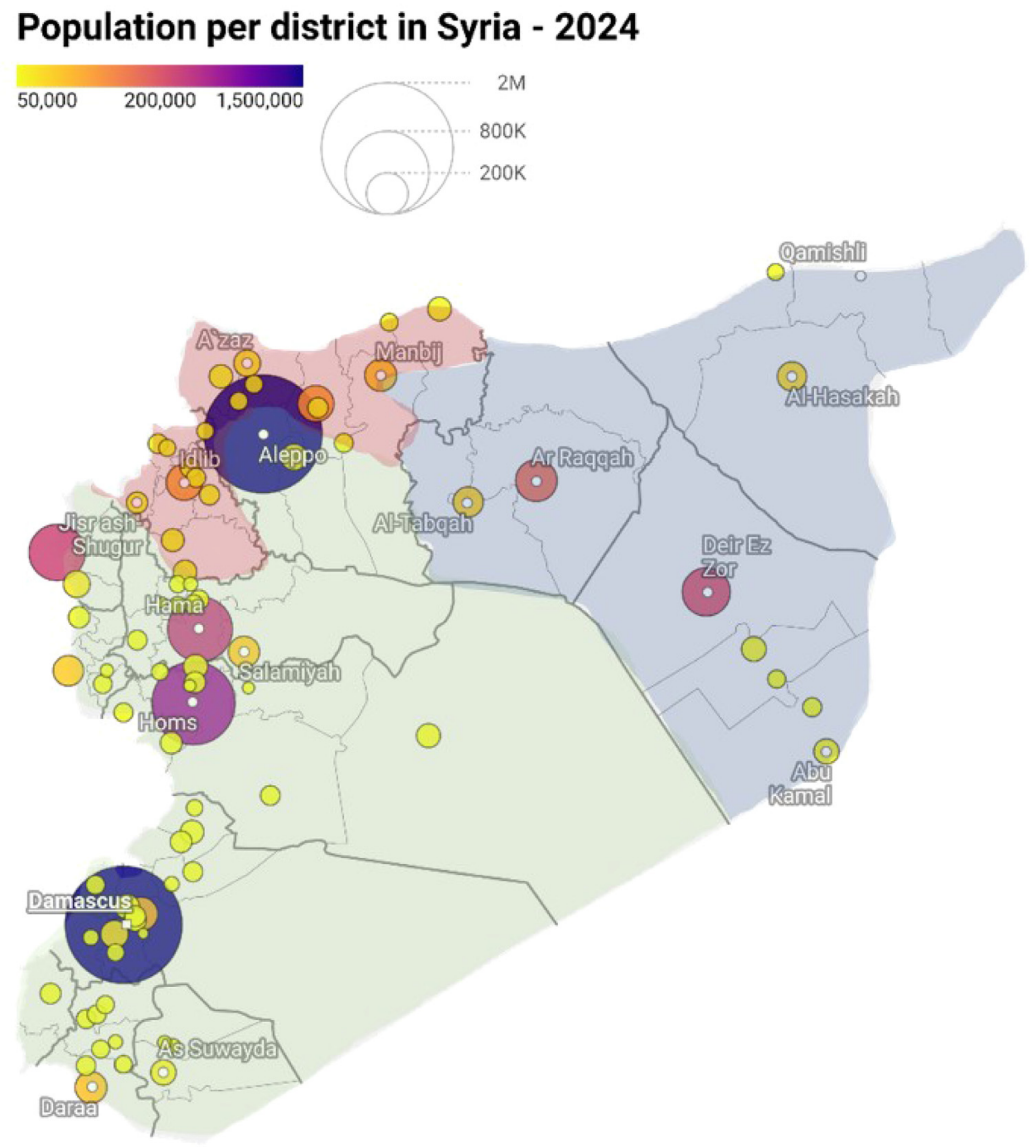
Population size and geographical coverage of the two Early Warning Systems in Syria until November 2024; Aleppo, and Damascus are the most populated cities. The areas shaded green are government-controlled areas with EWARS coverage and shaded blue (northeast Syria) and red (northwest Syria) with EWARN coverage. The map was created using Data Wrapper in June 2024.

NWS, which includes most of Idlib and parts of Aleppo governorates, has a population of 4.2-5 million people, approximately two-thirds of whom are internally displaced [32]. Moreover, NES contains an estimated 3 million people and includes the governorates of Al-Raqqa, Deir Ez-zor, Al-Hasakeh, and parts of rural Aleppo [26]. EWARN is functional in NWS and NES. EWARS operates in government-controlled areas (covering a population of 13 million) which have relatively more stable infrastructure and resources [11]. These two EWSs operate with limited communication. Figure 1 shows the population size and the EWARS-EWARN geographical coverage.

### Data collection

EWARS data were sourced from publicly available WHO reports covering government-controlled areas in Syria. For EWARN, the data were acquired through a data-sharing request with the ACU. These data sets include detailed information on various syndromes and diseases under surveillance (e.g. measles, ABD, AJS, and SARI), case counts, sentinel sites, completeness, and timeliness. In addition, the ACU provided data on alerts and confirmed outbreaks by district and disease/syndrome. Table 1 summarizes the epidemiologic metrics used in this study.

**Table 1:** Definition and calculation of the epidemiological metrics used in the study.

Metrics	Definition	Method of calculation
**Timeliness**	Number of reporting sites meeting the weekly reporting deadline. Presented as a percentage	(Number of sites meeting the deadline ÷ total number of reporting sites) × 100
**Completeness**	Number of reporting sites producing a complete dataset. Presented as a percentage	(Number of sites producing a complete dataset ÷ total number of reporting sites) × 100
**Coverage**	Geographical coverage of the surveillance systems.	-
**Sensitivity**	The ability of the alert threshold to correctly identify true outbreak events. Presented as a percentage	$\mathrm{Sens}\mathrm{itiv}\mathrm{ity}=\frac{\mathrm{True}\;\mathrm{Posi}\mathrm{tives}}{\mathrm{True}\;\mathrm{Posi}\mathrm{tives}+\mathrm{False}\;\mathrm{Nega}\mathrm{tives}}\times 100$
**Specificity**	The ability of the alert threshold to correctly identify non-outbreak events. Presented as a percentage	$Specificity=\frac{True\;Negatives}{True\;Negatives+False\;Positives}\times 100$
**Youden index**	A metric that ranges between 0 and 1 and maximizes the difference between true positive and false positive rates, used to identify the optimal threshold. Results closer to 1 indicate better performance.	$Youden^{\prime }s\;Index=Sensitivity+Specificity-1$
**Area under the curve**	A measure of the overall performance of a diagnostic test, indicating how well the test can distinguish between outbreak and non-outbreak periods. AUC values range from 0.5 (no discrimination) to 1 (perfect discrimination).	Calculated from the receiver operating characteristic (ROC) curve, which plots the true positive rate against the false positive rate across various thresholds.
**Lag window**	The number of weeks between the first alert generated by the tested threshold and the peak in the number of cases, indicating how early the system can detect an impending outbreak.	The number of weeks between the first alert and peak of number of cases
**Percentile method (P_x_)**	The threshold is calculated as the x percentile of the historical data for the same epidemiologic week of interest. Where x can be the 75^th^, 80^th^, 85^th^, or 90^th^ percentile of the historical data.	$\mathrm{Thre}\mathrm{shold}=\mathrm{Perc}\mathrm{enti}\mathrm{le}\;\mathrm{value}\;(P_{x})\;\mathrm{of}\;\mathrm{hist}\mathrm{oric}\mathrm{al}\;\mathrm{cases}$ Where x = 75, 80, 85, and 90
**Cumulative sum (CUMSUM)**	The threshold is calculated based on the mean and SD of 3 weeks (the week of interest and previous 2 weeks).	Threshold=Mean+2×StandardDeviation
**Historical average (HISAVG)**	The threshold is calculated based on the average value for 5 weeks (the week of interest, 2 weeks before, and 2 weeks after), using the historical data in the previous years for the selected weeks.	$Threshold=\left(\frac{\sum_{i=1}^{n}\left(Average\;of\;5\;weeks\;per\;year_{i}\right)}{n}\right)$ Where n = number of years included

### Data analysis

#### Descriptive analysis

**Figure 2:**
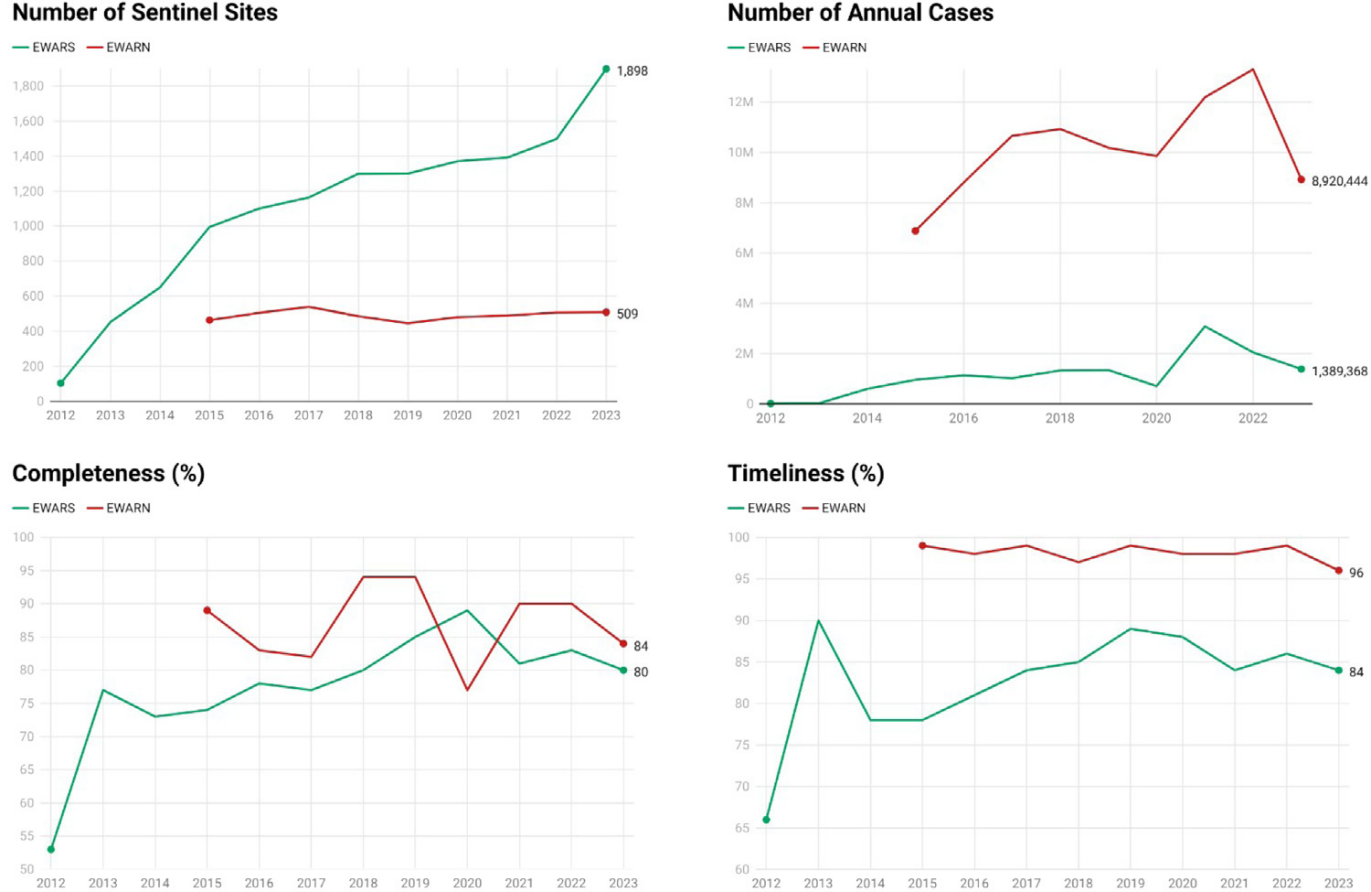
Comparison of EWARS and EWARN between 2012 and 2023 regarding the number of sentinel sites, number of annual cases, completeness, and timeliness.

A descriptive analysis was performed to calculate the completeness (the percentage of reporting sites producing a complete data set) and timeliness (the percentage of reporting sites meeting the weekly reporting deadline) of EWARN and EWARS (Figure 2). Moreover, the total number of cases per year and the number of sentinel sites were reported (Figure 2).

The descriptive analysis also showed the number of alerts (the number of cases crossing a specific alert threshold) and outbreaks (confirmed outbreaks based on epidemiologic investigation methods) per disease and district, as recorded by EWARN. Diseases and districts with the highest number of alerts were used in the second stage of the analysis. Detailed tables (Annex 1 and Annex 2 in the appendix) present the number of alerts per disease and district that informed this selection.

#### Adjusting the alert threshold

The process of testing and adjusting the alert thresholds for different diseases aims to improve the system's ability to detect outbreaks accurately while minimizing false alerts. This process has been applied to four diseases: measles, ABD, AJS, and SARI. Measles was included because of its high public health risk, particularly in conflict areas, and the current threshold was set at five cases per 100,000 people per week per district. ABD, AJS, and SARI were included because they had the highest number of cases and frequently exceeded the standard alert thresholds (**Annex 1**). For these diseases, the EWARN threshold was set to twice the average of the previous 3 weeks. The analysis was based on 2023 data because alert and confirmed outbreak information were available.

The analysis was conducted on data from four districts—Azaz and Afrin (Aleppo), Harem (Idlib), and Ras al Ain (Al-Hasakah)—which experienced frequent surges in cases and were frequently highlighted in the confirmed outbreak database (**Annex 2**).

Statistical methods used included receiver operating characteristic (ROC) curves, Youden index, and area under the curve (AUC) calculations. ROC curves were used to assess the sensitivity (true positive rate) and specificity (true negative rate) of various thresholds. The Youden index was used to identify the optimal threshold or point at which sensitivity and specificity had the best balance. In this study, confirmed alerts were treated as true positives. The best threshold was selected based on the highest Youden index, AUC, and the longest lag time (weeks from the first alert to the peak number of cases). The AUC measured the overall ability of the threshold to distinguish between outbreak and non-outbreak periods, with higher values indicating better performance.

Three methods were applied according to the WHO guidelines [16,17]: the percentile method (using 75%, 80%, 85%, and 90% thresholds of historical data), the cumulative sum method (using the mean and SD of the current and previous 2 weeks), and the historical average approach (using case counts from the same week in previous years). These methods aim to enhance the outbreak detection accuracy by adjusting the thresholds accordingly. The specific formulas for calculating each threshold are listed in Table 1.

## Results

A total of 104,926,159 cases were reported during the study period. The annual average number of reported cases was 1,140,717 for EWARS and 10,189,415 for EWARN. The geographical and population coverage of each EWS has changed over time, but relative stability has been observed since 2019. The results of this study indicate an increase in the number of sentinel sites for EWARS and EWARN from 2012 to 2023 (Figure 2). EWARS showed a steady increase in the number of sites, from 104 in 2012 to 1,898 in 2023. Conversely, EWARN has exhibited fluctuations in the number of sites since its initiation in 2015, reaching a peak in 2017 and stabilizing at 509 sites by 2023.

### Functional characteristics of EWARS/N

EWARS reported a substantial increase in the number of cases during the study period, with a notable surge in 2021 (3,085,401 cases). Although later in its initiation, EWARN reported a higher overall case count, peaking in 2022 (13,301,555 cases). The completeness of reporting for both systems varied. EWARS demonstrated an upward trend, peaking at 89.67% in 2020, with a slight decrease in 2023. EWARN showed initially high completeness that fluctuated in subsequent years, dipping in 2020, but recovering to 84% by 2023. In terms of timeliness, EWARS and EWARN maintained relatively consistent levels (Figure 2).

Since its inception in 2012, EWARS has reported cases in all Syrian governorates. In contrast, EWARN's coverage has undergone changes over the years because frontlines and access during the conflict have changed. In 2015, EWARN covered 12 of 14 Syrian governorates, excluding Al-Swedaa and Tartous. By 2019, because the conflict lines changed and the government took more area from opposition forces, coverage was reduced to six governorates. Since 2020, EWARN has actively covered all or parts of five governorates: Aleppo, Al-Hasakah, Al-Raqqa, Deir Ez-zor, and Idlib.

### Adjusting the alert threshold

The current alert thresholds for different diseases resulted in 574 alerts between 2018 and 2024 from the five governorates under EWARN surveillance. The measles alert threshold was crossed 25 times, ABD 47 times, AJS 111 times, and SARI 87 times (**Annex 1)**.

For measles, the 85^th^ percentile method provided a balanced approach, with a sensitivity of 81% and specificity of 63%, and had the highest Youden index of 0.443 compared with other methods, the highest AUC (0.793), and a lag window of 2 weeks. Other methods, such as 75^th^ percentile and historical average method, have higher sensitivities but lower specificities (Table 2).

**Table 2:** Sensitivity and specificity of the tested threshold.

Infectious diseases	Current alert threshold	Adjusted threshold	Sensitivity (%)	Specificity (%)	Youden index	Area under the curve	Lag window (weeks)
**Measles**	**>5 cases per 100.000 population per district**	P75	95	41	.361	.697	7
		P80	81	48	.292	.692	3
		**P85**	**81**	**63**	**.443**	**.793**	**2**
		P90	52	78	.330	.749	0
		CUMSUM	10	86	0	.567	0
		HISAVG	95	28	.233	.748	9
**ABD**	**Double the average of the previous 3 weeks (sum of cases)**	**P75**	**71**	**82**	**.532**	**.796**	8
		P80	22	85	.073	.591	6
		P85	22	89	.112	.594	2
		P90	22	96	.181	.595	1
		CUMSUM	28	90	.182	.519	0
		HISAVG	56	70	.263	.631	9
**AJS**	**Double the average of the previous 3 weeks (sum of cases)**	**P75**	**29**	**85**	**.145**	**.503**	15
		P80	18	85	.038	.411	9
		P85	15	85	0	.372	2
		P90	13	89	.028	.358	1
		CUMSUM	21	83	.047	.414	0
		HISAVG	32	74	.065	.421	18
**SARI**	**Double the average of the previous 3 weeks (sum of cases)**	P75	75	56	.315	.779	23
		P80	75	63	.386	.779	20
		P85	75	69	.447	.833	11
		**P90**	**75**	**90**	**.653**	**.900**	2
		CUMSUM	50	82	.322	.702	0
		HISAVG	75	49	.244	.817	28

Px: percentile method, where x = 75%, 80%, 85%, and 90%.

CUMSUM, cumulative sum method; HISAVG, historical average method.

For ABD, the 75^th^ percentile had the highest Youden index (0.532), AUC (0.796), and longest lag window, whereas for AJS, the 75^th^ percentile had the best results; however, the results of all methods were low and did not indicate good sensitivity (Table 2).

Finally, for SARI, the 90^th^ percentile method had the best overall results and presented a 2 week lag window, whereas adjusting to the 85^th^ percentile showed a slight decrease in sensitivity and specificity but provided a longer lag window of 11 weeks (Table 2).

**Figure 3:**
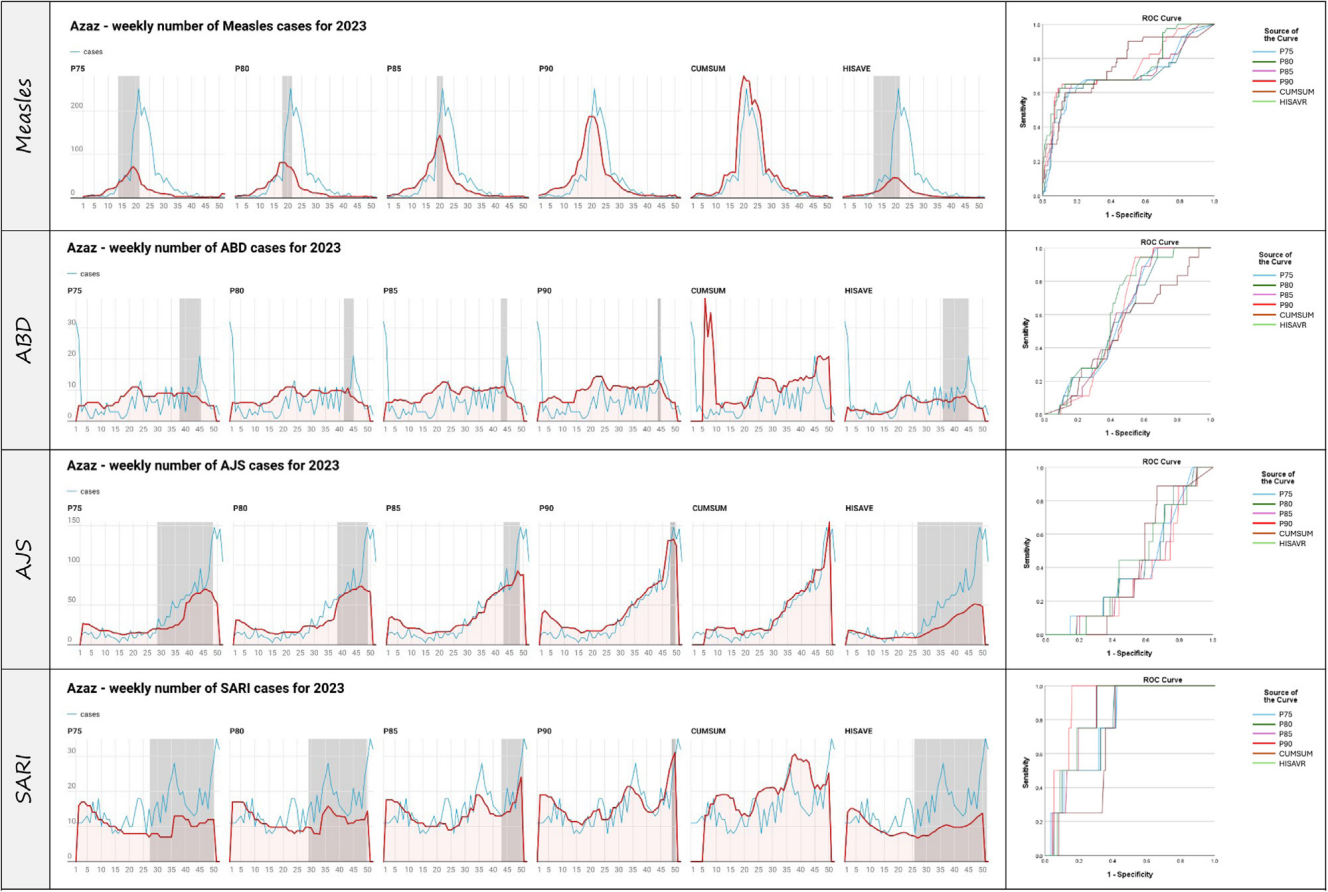
Visual representation of the tested threshold against the four diseases in Azaz district 2023, where the red line represents the threshold produced using different method, the blue line represents the total number of cases, and the gray area represents the lag window between the first alert and the peak of the cases. Also, an ROC curve was produced for each of the thresholds. ABD, acute watery diarrhea; AJS, acute jaundice syndrome; ROC, receiver operating characteristic; SARI, severe acute respiratory infection.

Figure 3 presents a visualization of the total number of cases for measles, ABD, AJS, and SARI in Azaz district for 2023, along with a visualization of the adjusted threshold for the same period. The gray area highlighted on the epidemiologic curve represents the lag window in weeks between the first alert until the peak number of cases. The ROC curve represents the sensitivity and specificity for each of the thresholds tested in this study and visualizes the AUC. Azaz was chosen as a case study to visualize the cases and thresholds because of the high number of cases and several recorded alerts (Annex 2).

## Discussion

This study was based on the changing geopolitical and epidemiologic situation in Syria, where two separate EWSs, EWARN and EWARS, operate using different methods, regional focuses, and disease monitoring priorities. Together, they cover the entirety of Syria's governorates, with some areas experiencing overlapping surveillance. However, despite this seemingly broad coverage, multiple infectious disease outbreaks occurred and spread during the Syrian crisis. This raises important questions regarding how well these systems function and their effectiveness in alerting, controlling, and responding to public health threats. This study aimed to assess different alert thresholds that may offer improved alternatives to those currently in use.

Analysis of the functional characteristics showed stability in some respects and improvements in others, particularly the continuous expansion in the number of sentinel sites for EWARS compared with previous years [21]. EWARN continues to report higher case numbers than EWARS, despite covering a smaller area and population. This could be because of the complexities of working in conflict zones, such as case duplication from overlapping surveillance and difficulties in verifying data. However, it may also reflect a true case burden given the high levels of conflict and displacement in northern Syria and EWARN's improved access to the area. EWARS may also be underreporting in areas outside government control owing to limited access and operational constraints. No major changes were observed in the completeness, timeliness, or number of cases compared with previous years for EWARN and EWARS [21].

The results of this study indicate that alert thresholds should be tailored for each disease rather than applying a single threshold across many diseases. This was evident from the findings, which showed that the optimal alert threshold varied among the selected diseases. In addition, selecting an appropriate threshold is complex because it involves balancing sensitivity, specificity, and lag time [28,29]. Lag time is particularly crucial because it affects the preparation and implementation of public health interventions, which can vary between different diseases. Therefore, adjusting the alert thresholds for each disease can enhance early outbreak detection and ensure timely and effective public health responses [29].

Our findings align with those of Rguig et al. [28] in Morocco, who emphasized the necessity of disease-specific alert thresholds to enhance early outbreak detection. Similar to Rguig et al.’s optimization of influenza thresholds, our results demonstrate that a one-size-fits-all approach is inadequate because optimal thresholds vary significantly across different diseases [28]. This reinforces the importance of customizing thresholds for specific epidemiologic patterns of each disease to improve the sensitivity and specificity of EWSs. Likewise, Wang et al.’s [29,30] research on the CIDARS supports this approach by showing that tailored thresholds using advanced algorithms significantly enhance the detection accuracy for various infectious diseases, further validating the concept of disease-specific optimization as a critical strategy for effective outbreak detection and response.

However, our study also highlights the challenges that differentiate the Syrian EWSs from the more structured approaches observed in other contexts. Although Wang et al. [29,30] were able to refine the thresholds within a highly controlled and resource-abundant setting in China, our research had to adapt to the resource-limited environment of Syria. This complicates the threshold adjustment process because it requires balancing sensitivity, specificity, and lag time while accounting for logistical and operational constraints that can delay public health interventions. This complexity was also observed in the work of Nagbe et al. [33] in Liberia, where operational limitations often impeded timely responses, even when thresholds were met.

This study had several limitations. First, the EWARN system operates in a challenging, under-resourced context, which affects the data quality. The analysis was limited to 2023 data, potentially missing variability in outbreak trends from previous years, which could have biased the results. In addition, implementing the proposed thresholds in low-resource settings poses challenges owing to limited capacity at the data collection and processing levels. Despite these limitations, this study provides a methodological foundation for developing alert thresholds based on statistical methods rather than relying on personal experience or expert judgment, which is currently done with EWARN. This study lays the foundation for future studies to refine and optimize alert thresholds across various diseases and settings, enhance the functionality of EWSs, and improve global public health responses, particularly in conflict-affected settings. Future investigations should focus on improving data quality and testing different thresholds to further strengthen outbreak detection capabilities. Despite the fall of the regime in December 2024 and the likely merger of the EWSs in Syria or the formation of a more formal surveillance system, findings from this study remain useful as a means of informating future systems.

## Conclusion

Syria presents a unique situation with two independent EWSs (EWARN and EWARS), highlighting the need for continuous evaluation and improvement of these systems. This study supports the idea that different diseases require different optimal thresholds, underscoring the fact that a single-threshold approach is inadequate. The percentile method demonstrated promising results and could be adjusted to improve the sensitivity, specificity, and lag time based on the situation and number of cases. Further research is required to develop and validate more sophisticated statistical methods for setting disease-specific thresholds that can be applied across various EWSs in different contexts.

## Declarations of competing interest

The authors have no competing interests to declare.

## Appendix Supplementary materials

Image, application 1

Image, application 2
