# Supplementary material for: The early warning and response systems in Syria: A functionality and alert threshold assessment
Source: IJID Reg. 2025 Jan 7;14:100563. doi: 10.1016/j.ijregi.2024.100563 (PMC11795629; doi:10.1016/j.ijregi.2024.100563)
Supplement: Supplementary file 1 [file mmc1.docx]

***Annex 1. EWARN alert and confirmed outbreak for each of the diseases under surveillance between 2018-2024***

|  | 2018 | | 2019 | | 2020 | | 2021 | | 2022 | | 2023 | | 2024 | | Grand Total |
| --- | --- | --- | --- | --- | --- | --- | --- | --- | --- | --- | --- | --- | --- | --- | --- |
| Disease/syndromes | **Alert** | **Outbreak** | **Alert** | **Outbreak** | **Alert** | **Outbreak** | **Alert** | **Outbreak** | **Alert** | **Outbreak** | **Alert** | **Outbreak** | **Alert** | **Outbreak** |  |
| ABD | 1 | 1 | 9 |  | 6 |  | 4 |  | 16 |  | 10 |  | 1 |  | 48 |
| AFP |  |  |  |  |  |  |  |  | 1 |  |  |  |  |  | 1 |
| AJS | 18 | 4 | 3 |  | 22 |  | 17 |  | 13 |  | 35 |  | 3 |  | 115 |
| AWD | 3 |  | 2 |  | 2 |  | 1 |  | 36 | 1 | 4 |  |  |  | 49 |
| Chickenpox |  | 1 | 2 | 1 |  |  |  |  |  |  |  |  |  |  | 4 |
| Food Poisoninig | 2 |  |  |  |  |  |  |  |  |  |  |  |  |  | 2 |
| ILI | 3 |  |  |  | 5 |  |  |  | 2 |  | 6 |  |  |  | 16 |
| Impetigo |  |  |  | 1 |  |  |  |  |  |  |  |  |  |  | 1 |
| LEISH |  | 1 | 7 | 2 | 4 |  | 3 | 2 | 2 |  |  |  | 3 |  | 24 |
| Lice |  | 8 | 2 | 1 |  |  |  |  |  |  |  |  |  |  | 11 |
| MEA |  |  |  |  |  |  |  |  | 19 | 1 | 6 |  |  |  | 26 |
| MEN | 2 | 1 | 5 |  | 3 |  | 1 |  | 8 | 1 | 6 |  |  |  | 27 |
| Mumps |  | 2 | 2 | 4 |  |  |  |  |  |  |  |  |  |  | 8 |
| OAD | 3 | 6 | 9 | 1 | 6 | 3 | 9 | 3 | 4 |  | 2 |  | 1 | 1 | 48 |
| Others |  |  |  |  | 8 | 6 | 23 | 2 | 29 | 10 | 28 | 3 | 7 | 2 | 118 |
| Pertussis |  |  | 2 |  |  |  |  |  |  |  |  |  |  |  | 2 |
| SARI | 9 | 2 | 8 |  | 24 |  | 11 |  | 17 | 1 | 14 |  | 4 |  | 90 |
| Scabies |  | 3 | 1 | 2 |  |  |  |  |  |  |  |  |  |  | 6 |
| STF | 10 | 11 | 5 | 1 | 8 |  | 3 |  | 9 |  | 6 |  |  |  | 53 |
| UCE |  |  | 3 | 1 | 2 |  |  |  |  |  | 3 |  | 5 |  | 14 |
| UXD |  |  |  |  |  |  | 1 |  |  |  |  |  |  |  | 1 |
| Grand Total | **51** | **40** | **60** | **14** | **90** | **9** | **73** | **7** | **156** | **14** | **120** | **3** | **24** | **3** | **664** |
