# Supplementary material for: The early warning and response systems in Syria: A functionality and alert threshold assessment
Source: IJID Reg. 2025 Jan 7;14:100563. doi: 10.1016/j.ijregi.2024.100563 (PMC11795629; doi:10.1016/j.ijregi.2024.100563)
Supplement: Supplementary file 2 [file mmc2.docx]

***Annex 2. EWARN alert and confirmed outbreak for each of the districts under coverage between 2018-2024***

|  | 2018 | | 2019 | | 2020 | | 2021 | | 2022 | | 2023 | | 2024 | | Grand Total |
| --- | --- | --- | --- | --- | --- | --- | --- | --- | --- | --- | --- | --- | --- | --- | --- |
| Districts | **Alert** | **Outbreak** | **Alert** | **Outbreak** | **Alert** | **Outbreak** | **Alert** | **Outbreak** | **Alert** | **Outbreak** | **Alert** | **Outbreak** | **Alert** | **Outbreak** |  |
| Abu Kamal |  |  |  |  |  |  |  |  |  |  | 1 |  |  |  | 1 |
| Afrin | 3 | 1 | 1 |  | 10 | 2 | 6 |  | 32 |  | 15 |  | 6 | 2 | 78 |
| Ain Al Arab |  |  |  |  |  |  |  |  | 1 |  | 2 |  |  |  | 3 |
| Al Bab | 9 | 8 |  | 1 | 3 | 1 | 2 |  | 4 | 1 |  |  | 2 |  | 31 |
| Al Ma'ra | 2 | 4 |  |  |  |  |  |  |  |  |  |  |  |  | 6 |
| Al Mayadin |  |  |  | 1 |  |  |  |  | 1 |  |  |  |  |  | 2 |
| Al-Hasakeh | 1 |  |  |  | 1 |  | 6 |  | 5 |  | 4 |  | 1 |  | 18 |
| Al-Malikeyyeh |  | 1 |  |  |  |  | 4 |  | 8 |  | 2 |  | 3 |  | 18 |
| Ariha |  | 1 | 1 |  | 1 |  |  |  | 1 |  | 1 |  | 1 |  | 6 |
| Al-Raqqa | 6 | 4 | 7 | 1 | 6 | 1 | 9 |  | 6 | 3 |  |  |  |  | 43 |
| As-Sanamayn | 1 |  |  |  |  |  |  |  |  |  |  |  |  |  | 1 |
| Ath-Thawrah | 3 | 2 | 1 | 1 | 3 |  | 3 |  | 5 |  |  | 1 |  |  | 19 |
| A'zaz | 2 |  | 6 | 2 | 6 |  | 1 |  | 13 |  | 13 |  |  |  | 43 |
| Dar'a | 2 |  |  |  |  |  |  |  |  |  |  |  |  |  | 2 |
| Deir Ez-Zor |  | 2 |  |  | 6 |  | 2 |  | 4 |  | 3 |  |  |  | 17 |
| Hama |  | 1 |  |  |  |  |  |  |  |  |  |  |  |  | 1 |
| Harim | 4 | 7 | 13 | 3 | 13 | 2 | 9 | 2 | 25 | 2 | 27 | 2 | 2 |  | 111 |
| Idleb | 4 | 4 | 3 | 2 | 7 | 3 | 2 | 2 | 14 | 3 | 12 |  | 1 |  | 57 |
| Jarablus | 1 |  | 6 | 1 | 8 |  | 6 |  | 11 |  | 4 |  | 4 | 1 | 42 |
| Jebel Saman | 8 | 2 | 7 | 1 | 7 |  | 4 |  | 3 |  | 17 |  | 1 |  | 50 |
| Jisr-Ash-Shugur | 3 |  | 2 |  | 6 |  | 1 |  | 2 | 2 | 9 |  |  |  | 25 |
| Menbij |  |  | 2 |  |  |  |  |  | 1 |  |  |  |  |  | 3 |
| Quamishli | 2 | 2 | 11 |  | 5 |  | 6 |  | 5 |  | 4 |  | 2 |  | 37 |
| Ras Al Ain |  |  |  |  | 3 |  | 10 | 2 | 8 | 3 | 3 |  | 1 |  | 30 |
| Rural Damascus |  | 1 |  |  |  |  |  |  |  |  |  |  |  |  | 1 |
| Tell Abiad |  |  |  | 1 | 5 |  | 2 | 1 | 7 |  | 3 |  |  |  | 19 |
| Grand Total | **51** | **40** | **60** | **14** | **90** | **9** | **73** | **7** | **156** | **14** | **120** | **3** | **24** | **3** | **664** |
